# Retinal degeneration protein 3 controls membrane guanylate cyclase activities in brain tissue

**DOI:** 10.3389/fnmol.2022.1076430

**Published:** 2022-12-21

**Authors:** Yaoyu Chen, Anja U. Bräuer, Karl-Wilhelm Koch

**Affiliations:** ^1^Division of Biochemistry, Department of Neuroscience, Carl von Ossietzky University, Oldenburg, Germany; ^2^Division of Anatomy, Department of Human Medicine, Carl von Ossietzky University, Oldenburg, Germany; ^3^Research Center Neurosensory Science, Carl von Ossietzky University, Oldenburg, Germany

**Keywords:** natriuretic peptide, membrane bound guanylate cyclase, GC-A, GC-B, RD3 protein

## Abstract

The retinal degeneration protein RD3 is involved in regulatory processes of photoreceptor cells. Among its main functions is the inhibition of photoreceptor specific membrane guanylate cyclases during trafficking from the inner segment to their final destination in the outer segment. However, any physiological role of RD3 in non-retinal tissue is unsolved at present and specific protein targets outside of retinal tissue have not been identified so far. The family of membrane bound guanylate cyclases share a high homology of their amino acid sequences in their cytoplasmic domains. Therefore, we reasoned that membrane guanylate cyclases that are activated by natriuretic peptides are also regulated by RD3. We analyzed transcript levels of the *rd3* gene and natriuretic peptide receptor genes *Npr1* and *Npr2* in the mouse retina, cerebellum, hippocampus, neocortex, and the olfactory bulb during development from the embryonic to the postnatal stage at P60. The *rd3* gene showed a lower expression level than *Npr1* and *Npr2* (encoding for GC-A and GC-B, respectively) in all tested brain tissues, but was at least one order of magnitude higher in the retina. RD3 and natriuretic peptide receptor GCs co-express in the retina and brain tissue leading to functional tests. We expressed GC-A and GC-B in HEK293T cells and measured the inhibition of GCs by RD3 after activation by natriuretic peptides yielding inhibitory constants around 25 nM. Furthermore, endogenous GCs in astrocytes were inhibited by RD3 to a similar extent. We here show for the first time that RD3 can inhibit two hormone-stimulated GCs, namely GC-A and GC-B indicating a new regulatory feature of these hormone receptors.

## Introduction

Membrane bound guanylate cyclases (GC) synthesize the second messenger guanosine 3′,5′-cyclic monophosphate (cGMP) that is involved in a variety of physiological functions including blood pressure, skeletal growth, kidney function, phototransduction, olfaction, and thermosensation (Sharma, 2010; Koch and Dell’Orco, 2015; Kuhn, 2016; Sharma et al., 2016; Pandey, 2021). Extracellular ligands like natriuretic peptides (ANP, BNP, and CNP) bind to natriuretic peptide receptors at their extracellular ligand domains. These natriuretic peptide receptors are GCs switching to the active state, when binding natriuretic peptides. ANP and BNP activate GC-A expressed in the cardiovascular system and metabolic organs being involved in the regulation of blood pressure and metabolism. CNP activates GC-B and the CNP/GC-B signaling system is expressed in various organs, but also in cardiovascular cell types (Pandey, 2021). One of its main suggested physiological roles is to regulate skeletal growth (Kuhn, 2016). Expression of CNP and GC-B in different brain regions also indicate a functional role in neuronal tissue. For example, studies investigated a role in synaptic plasticity in the hippocampus (Decker et al., 2010; Barmashenko et al., 2014) and an involvement in the regulation of the circadian clock (Olcese et al., 2002). Natriuretic peptides and their receptors have also been identified in the retina (Rollín et al., 2004) and were linked to dopaminergic and cholinergic signaling in amacrine cells (Abdelalim et al., 2008), and to the modulation of GABA-receptor activity in bipolar cells (Yu et al., 2006). In addition to neuronal signaling, natriuretic peptides ANP and BNP exert protective effects in retinal neovascularization (Špiranec Spes et al., 2020).

A different activation process is found in membrane GCs expressed in sensory cells. For example, photoreceptor specific GC-E and GC-F form complexes with guanylate cyclase-activating proteins (GCAPs) on their intracellular site (Peshenko et al., 2011; Sulmann et al., 2017). GCAPs are calcium sensor proteins that respond to changes in cytoplasmic calcium concentration ([Ca^2+^]) with a conformational change thereby switching the target GC-E or GC-F to the active state (Koch and Dell'Orco, 2015; Dizhoor and Peshenko, 2021).

Point mutations in the genes *GUCY2D* and *GUCA1A* coding for GC-E and GCAP1 cause forms of retinal degeneration like cone-rod dystrophies. Leber’s congenital amaurosis (LCA) is a particularly severe form of retinal dysfunction leading to blindness after birth or in the first year of life. Patients suffering from LCA type 1 have point mutations in *GUCY2D* (Sharon et al., 2018) causing dysfunction of GC-E in alive photoreceptor cells opening routes for gene therapy (Jacobson et al., 2022). Mutations in the retinal degeneration protein 3 (RD3) of human patients correlate with the phenotypical characteristics of LCA12 (Friedman et al., 2006; Cideciyan 2010; Preising et al., 2012). In the physiologically normal state of a photoreceptor cell, RD3 binds to photoreceptor specific GCs and inhibits their GCAP-mediated activation at low cytoplasmic [Ca^2+^] (Peshenko et al., 2011, 2021). RD3 is further involved in GC-E trafficking from the endoplasmic reticulum to endosomal vesicles (Azadi et al., 2010; Molday et al., 2013; Zulliger et al., 2015). Correct trafficking and incorporation of GC-E in photoreceptor outer segments is essential for cell survival and interaction of RD3 with GC-E is a crucial requirement for these processes (Peshenko et al., 2016; Peshenko and Dizhoor, 2020; Plana-Bonamaisó et al., 2020). Wimberg et al. (2018a) observed in an *in vitro* study that purified RD3 evoked an increase in guanylate kinase (GUK) activity, an enzyme that is involved in the nucleotide cycle in photoreceptors catalyzing the 5’-GMP to GDP conversion. Both proteins directly interact and co-localize in photoreceptor inner segments and to a lesser extent in the outer plexiform layer in sections of the mouse retina. However, recent studies involving transgenic mice did not detect a regulatory impact of RD3 on GUK activity (Dizhoor et al., 2021).

Recent studies revealed that RD3 is also found in other tissue types such as epithelial cells and seems to be more ubiquitously expressed (Aravindan et al., 2017). Constitutive expression of RD3 was found in different mouse and human tissues including brain, kidney, liver, and spleen (Khan et al., 2015). The same authors further showed that RD3 loss in a mouse model correlates with aggressive neuroblastoma cancer. More recently, Somasundaram et al. (2019) found significant loss of RD3 expression on the transcript and protein level in patient derived tumor cells that survived intensive multi-modal clinical therapy. The authors conclude that transcriptional dysregulation might account for RD3 expression loss associated with advanced disease stage and low survival rate. However, these findings are in disagreement with a previous study showing that inactivation of both *rd3* alleles in LCA12 patients does not correlate with extraocular symptoms (Perrault et al., 2013).

The physiological role of RD3 in non-retinal tissue is unclear at present and basic issues related to its expression profile in brain tissue and the identity of its non-retinal targets are unsolved. A reasonable question in this context is, whether GCs activated by natriuretic peptide can be regulated by RD3. A necessary condition for such a regulation would be the expression of RD3 and GCs in the same tissue. We compared the expression levels of RD3, GC-A and GC-B in mouse retina and further determined their expression profiles in different brain regions during mouse brain development. We further compared gene expression in the retina with those in hippocampal neurons, astrocytes, and microglia. For functional studies, we expressed GC-A and GC-B in HEK293T cells and investigated the activity profiles in the presence and absence of added purified RD3.

## Materials and methods

### Heterologous expression of membrane GCs in HEK293T cell

HEK293T cells were grown in Dulbecco’s Modified Eagle Medium (DMEM; Thermo Fisher Scientific) supplemented with 10% fetal bovine serum (FBS; PAN-Biotech, Aidenbach, Germany), 2 mM L-glutamine (Merck Millipore, Darmstadt, Germany), 100 units/ml Penicillin–Streptomycin (PAN-Biotech) in the incubator set at 5% (v/v) CO_2_ and 37°C.

For cell transfection, the cDNA sequence of wildtype human GC-A and human GC-B was inserted into a pcDNA3.1 vector. The cDNA was amplified using the primers listed in the supplementary part (Supplementary Table S1). Cells were seeded for transfection in 100 mm petri dishes with 1.5 × 10^6^ cells per dish. After 24 h, cells were transfected with the pcDNA3.1 plasmids using the polyethylenimine (PEI) as transfection reagent. Stable cell lines expressing human GC-A or GC-B were created as described previously for GC-E (Wimberg et al., 2018a). The expression of GCs was confirmed *via* western blotting (see below) using commercial antibodies: polyclonal anti-GC-A (cat. Ab14357, Abcam) and polyclonal anti-GC-B (cat. Ab14356, Abcam). For the detection of photoreceptor GC-E we used a polyclonal antibody that was originally made against bovine GC-E, but showed crossreactivity to the human orthologue (Zägel et al., 2013). HA-tagged GC-A and GC-B were used in control experiments applying the pcDNA3.1 vector with HA-tag insertion.

### Expression and purification of RD3

The RD3 protein was expressed in *E.coli* and purified following the method as described before Wimberg et al., 2018a. Briefly, *E.coli* BL21+ cells containing the pET-M11-RD3-His6-tag construct was grown overnight in LB-Medium at 37°C after induction by 1 mM isopropyl-thiogalactoside (IPTG). The cell pellets were harvested by centrifugation and suspended in 50 mM Tris/HCl pH 8.0. Cells were lysed by adding 100 mg/ml lysozyme and 5 U/ml DNase and incubation at 30°C for 30 min. The lysate was centrifuged for 1.5 h at 50,000 × *g* at 4°C after adding 1 mM DTT and 0.1 mM phenylmethyl-sulfonylfluoride (PMSF). The RD3 containing pellet was homogenized in buffer 1 (20 mM phosphate buffer pH 7.4, 8 M urea, 10 mM imidazole, 500 mM NaCl, 5 mM β-mercaptoethanol (β-ME), 1 mM PMSF), and kept overnight at 4°C. The suspension was centrifuged for 1 h at 50,000 × *g* and 4°C, the supernatant was collected for Ni-NTA column-based purification. After sample loading onto the column, buffer 1 was applied for washing and was then gradually replaced by buffer 2 (20 mM phosphate buffer pH 7.4, 10 mM imidazole, 500 mM NaCl, 5 mM β-ME, 1 mM PMSF, 1 mM histidine, 10% glycerol) to facilitate protein refolding. Buffer 3 (20 mM phosphate buffer pH 7.4, 500 mM imidazole, 20 mM histidine, 500 mM NaCl, 5 mM β-ME, 1 mM PMSF) eluted RD3 containing fractions that were collected in volumes of 1 ml and analyzed *via* sodium dodecyl sulfate polyacrylamide gel electrophoresis (SDS-PAGE). Purified RD3 was tested for biological activity, namely inhibiting GCAP activated human GC-E present in HEK-293T cells that stably expressed GC-E (Wimberg et al., 2018a).

### Gel electrophoresis and immunoblotting

HEK-293T cells were harvested and the membrane protein fractions were collected as previously described Wimberg et al., 2018a. The protein fractions were incubated with 5 × Laemmli buffer containing 1% (v/v) β-ME at 95°C for 5 min and analyzed by SDS-PAGE having either 12% or 7.5% acrylamide. Immunoblotting was performed using a 0.45 μm nitrocellulose (NC) membrane, and a tank or semi-dry blotting system. RD3 was analyzed by SDS-PAGE on a 12% acrylamide gel and a semi-dry blotting procedure of 200 mA for 30 min. For GC-A and GC-B we used a 7.5% acrylamide gel and the tank blotting procedure (100 V for 70 min at 4°C). Afterwards, the blotted membrane was incubated with blocking solution [either 5% (w/v)] bovine serum albumin (BSA; Carl Roth, Karlsruhe, Germany, or 2.5–5% milk powder (Carl Roth)) in TBST at room temperature (RT) for 1 h. Primary anti-GC antibodies were incubated overnight at 4°C in blocking solution at a dilution of 1:5,000. The blot membranes were washed three-times with TBST at RT. Afterwards, the blot membranes were incubated for 1 h at RT with horseradish peroxidase-conjugated secondary antibodies (GE Healthcare, Boston, MA, United States) at a dilution of 1:10,000 in blocking solution. Membranes were again washed three times at RT with TBST, and immunoreaction was detected with Clarity or Clarity Max ECL substrate (Bio-Rad Laboratories, Hercules, CA, United States) according to the manufacturer’s protocol.

### Guanylate cyclase activity assay

The guanylate cyclase stimulator human atrial natriuretic factor (1–28; ANP, cat. H-2095) and the human c-type natriuretic peptide (1–53; CNP, cat. H-8420) were purchased from Bachem AG (Switzerland). Ten microliter of HEK-293T membranes expressing GC-A or GC-B were incubated with 2 μM ANP or CNP, or various concentrations RD3 in a final volume of 30 μl for 5 min at RT. The guanylate cyclase reaction was started by adding 20 μl GC buffer (75 mM Mops/KOH pH 7.2, 150 mM KCl, 10 mM NaCl, 2.5 mM DTT, 8.75 mM MgCl_2_, 4 mM GTP, 0.75 mM ATP, 0.4 mM Zaprinast). After incubation for 5 min at 30°C, the reaction was stopped by adding 50 μl 0.1 M EDTA and short incubation (5 min) at 95°C. After centrifugation at 13,000 × *g* for 10 min, the supernatant was harvested and analyzed for cGMP content as previously described (Wimberg et al., 2018a). Assays were pursued in three independent groups, each with 3 replicates (*N* = 9). Test series were performed to find the optimal assay conditions including a concentration dependence of GTP and a time series.

### RNA extraction, cDNA synthesis and qRT-PCR

The mRNA from mouse cerebellum, neocortex, hippocampus, and olfactory bulbs of the developmental stages of embryonic days (E) E14, E16, E19, and of postnatal days (P) P0, P5, P10, P15, P20, P30, P42, P60 was collected as described in Gross et al. (2022) and stored at-80°C until use. Briefly, the tissues were dissociated from sacrificed mice, at embryonic stages, one litter of all embryos (7–10 embryos; *N* = 21–30, sex not specified) were pooled. At postnatal stages, tissues from six mice of both sexes were pooled, for all stages with three independent replicates (*n* = 3). The retinas were obtained from the mice at P10, P20, P30 of both sexes with 3 replicates. Primary hippocampal neurons, astrocytes and microglia cells were obtained as described (Gross et al., 2022). The TRIzol™ reagent (Thermo Fisher Scientific, Waltham, MA, United States) was applied for RNA extraction from homogenized cells and tissues following the protocol from the manufacturer. RNA concentrations were determined by UV/Vis spectroscopy using the BioSpectrometer basic (Eppendorf, Hamburg, Germany). The cDNA was obtained according to the protocol of the high-capacity cDNA reverse transcription kit from Thermo Fisher Scientific.

Quantitative-RT-PCR was performed using the TaqMan™ Fast Universal PCR Master Mix, No AmpErase™ UNG (Thermo Fisher Scientific) on hard-shell 96-Well PCR plates from Bio-Rad Laboratories (Hercules, CA, United States), and TaqMan probes. Reactions were prepared according to the manufacturer’s protocols and detected by the CFX96 real-time PCR detection system (Bio-Rad Laboratories) using the following cycling parameters: 95°C for 20 s, 95°C for 1 s and 60°C for 20 s, for 45 cycles. Expression data of three independent preparations with duplicates of each reaction were calculated using the ΔCt method, with normalization to *Gapdh* and *Actb* as housekeeping genes.

### Culturing of astrocytes and GC activities in astrocytes

The astrocytes were cultured and collected according to Gross et al. (2022). Membranes containing membrane proteins were harvested after 10–12 days *in vitro* for endogenous guanylate cyclase activity tests. Guanylate cyclase activities in astrocyte membranes were stimulated by adding ANP or CNP. Inhibitory effects of RD3 were measured adding increasing amounts of purified RD3. The enzyme reaction started by adding 4 mM GTP. After 30 min incubation at 30°C, the reaction stopped by adding 0.1 M EDTA and incubation at 95°C for 5 min. The GC activities were measured using a cGMP ELISA Kit™ from Enzo Life Sciences according to the protocol of the manufacturer, three independent groups with two replicates each (*N* = 6). A cGMP standard curve was created between 0.01 and 500 pmol × mL^−1^ being maximally sensitive between 1 and 100 pmol × mL^−1^.

### Statistical analysis

**Figure 1:**
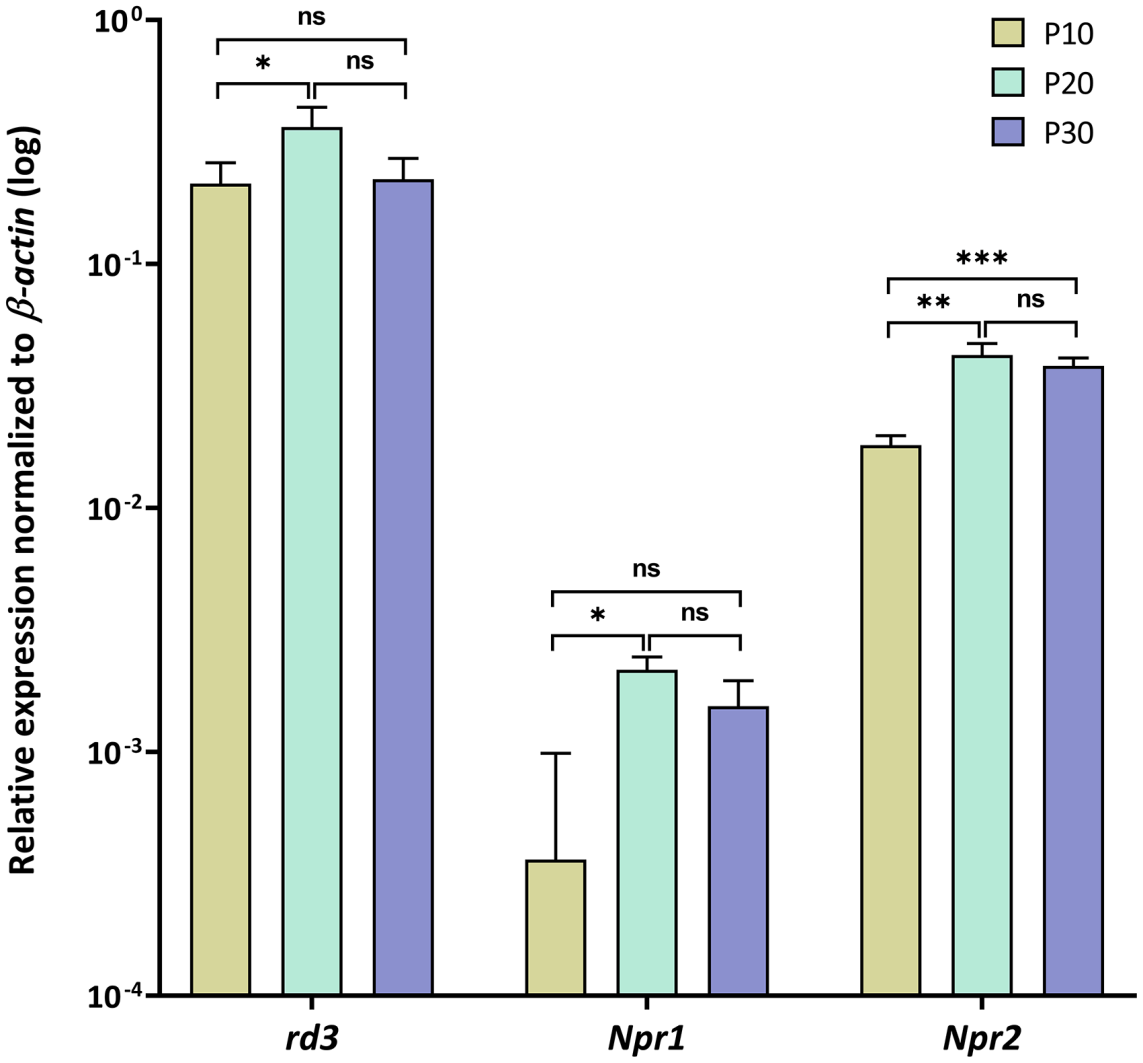
Gene expression profiles in mouse retina analyzed by quantitative RT-PCR. Relative transcript levels of *rd3*, *Npr1* and *Npr2* were determined in retina tissue at P10, P20 and P30 and normalized to β–*actin* expression. Analysis of statistics were based on one-way ANOVA followed by Bonferroni’s multiple comparisons test. Data are shown as mean ± SD. Specific numbers of *p*-values are shown in Supplementary Table S2 (Supplementary material).

**Figure 2:**
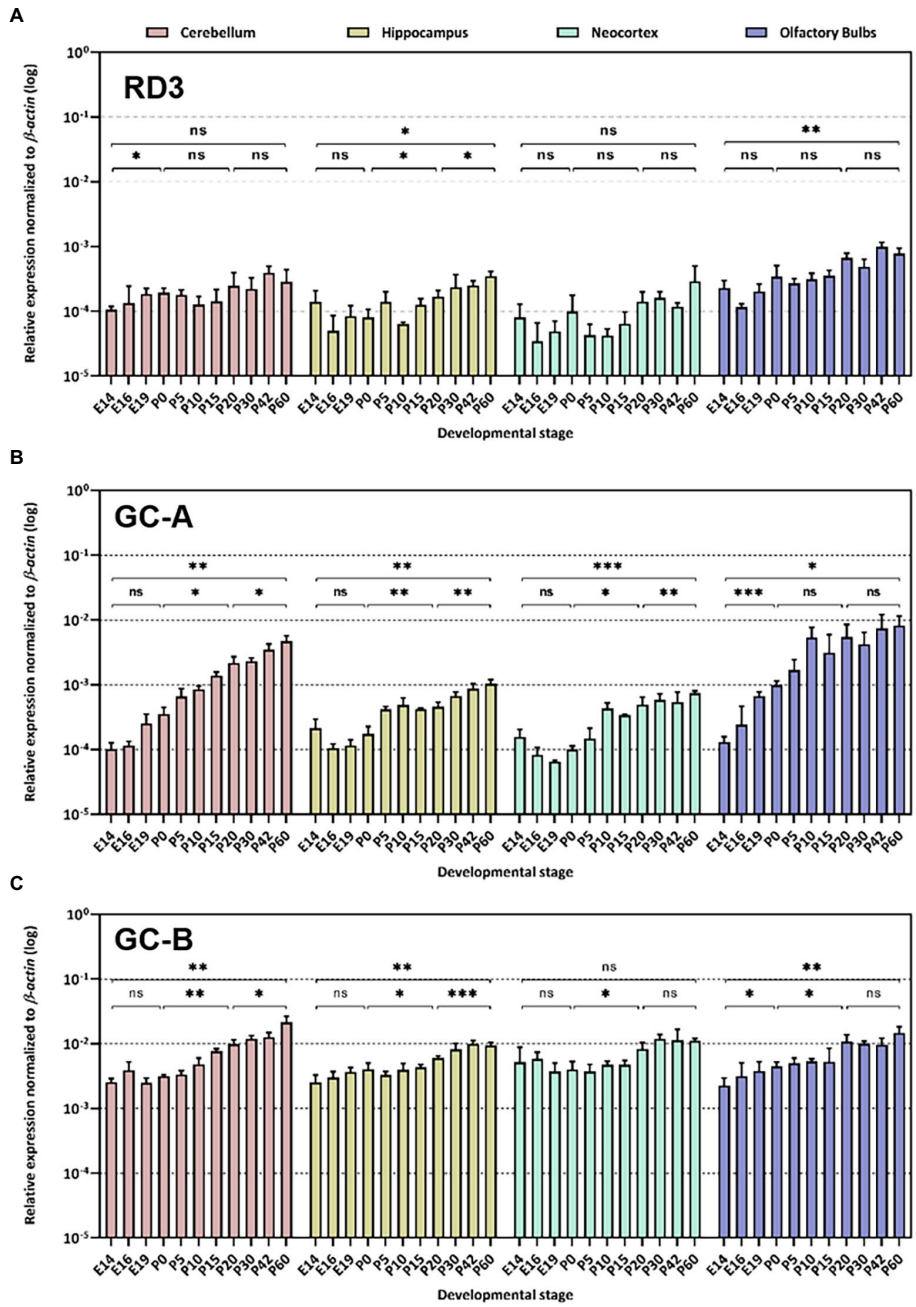
Quantitative RT-PCR. Gene expression pattern of *rd3* **(A)**, *Npr1* encoding GC-A **(B)** and *Npr2* encoding GC-B **(C)** was analyzed in different neuronal tissues as indicated. The relative expression was normalized to β–*actin* expression. Analysis of statistics were based on one-way ANOVA followed by Bonferroni’s multiple comparisons test. Data are shown as mean ± SD and were considered significant for *p* ≤ 0.05 (*p* ≤ 0.05 = *; *p* ≤ 0.01 = **; *p* ≤ 0.001 = ***; ns = not significant). Specific numbers of *p*-values are shown in Supplementary Table S3 (Supplementary material).

**Figure 3:**
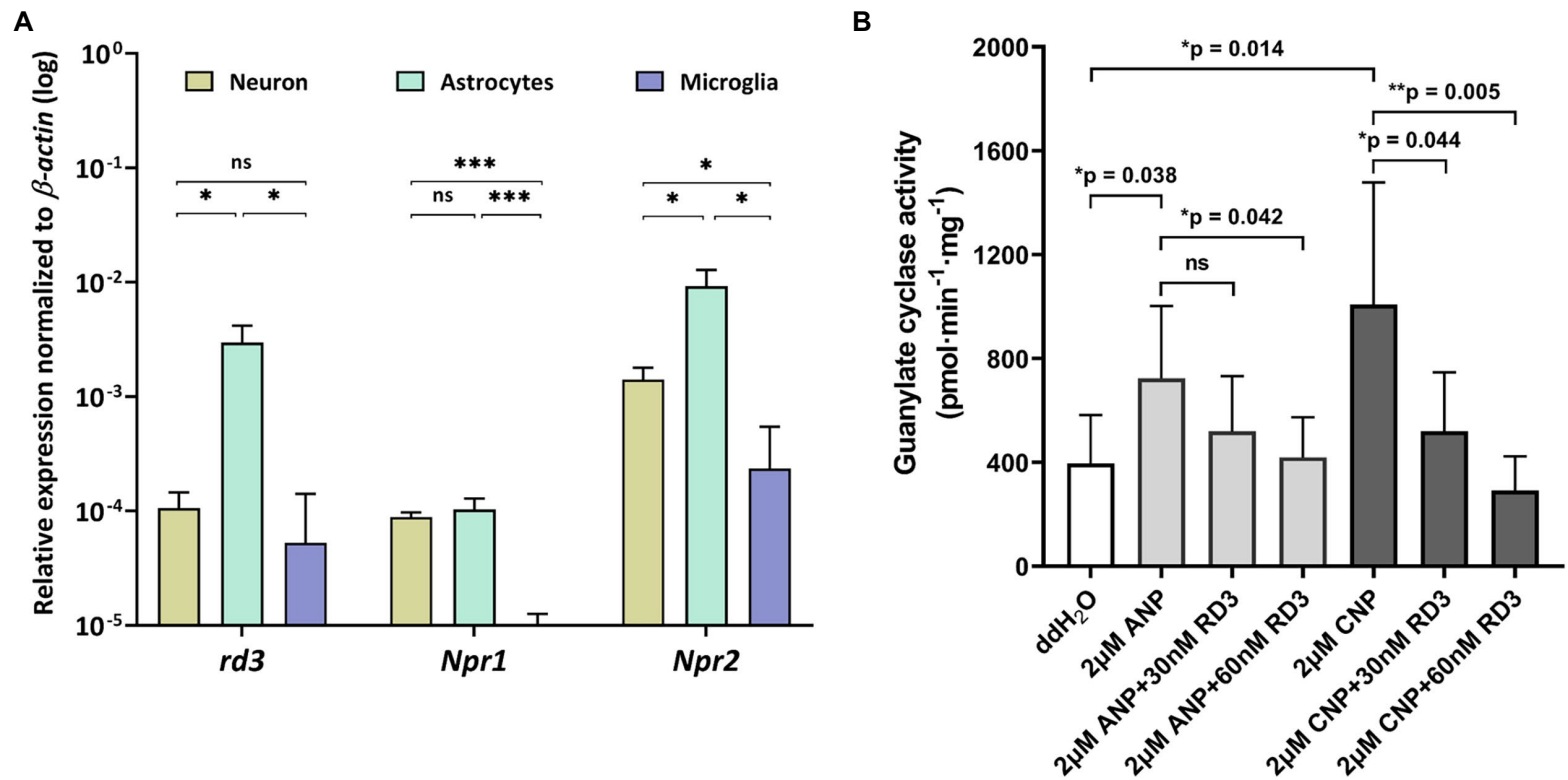
Expression and activity profiles of *rd3*, *Npr1* and *Npr2* in astrocytes. **(A)** Relative transcript levels of *rd3*, *Npr1* and *Npr2* in neurons, astrocytes, and microglia. Analysis of statistics were based on one-way ANOVA followed by Bonferroni’s multiple comparisons test. Data are shown as mean ± SD and were considered significant for *p* ≤ 0.05 (*p* ≤ 0.05 = *; *p* ≤ 0.01 = **; *p* ≤ 0.001 = ***; ns = not significant). Specific numbers of *p*-values are shown in Supplementary Table S4 (Supplementary material). **(B)** Inhibition of GC-A and GC-B in astrocytes by RD3.

**Figure 4:**
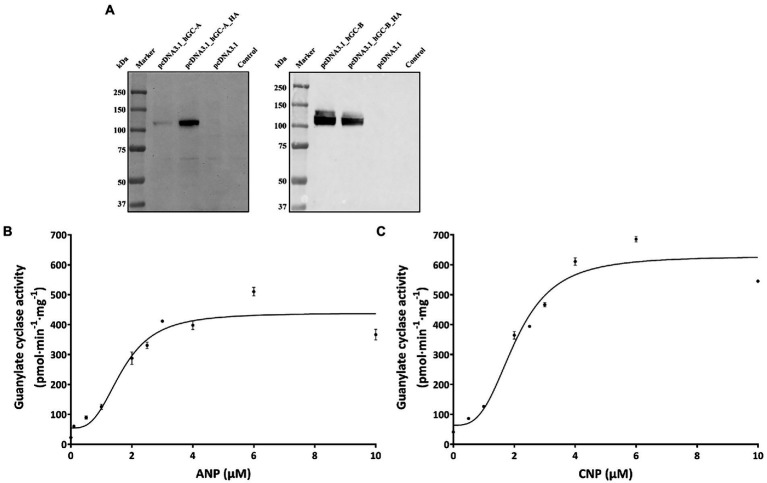
Heterologous expression GC-A and GC-B in HEK 293 T cells. **(A)** Immunoblotting of HEK 293 T cell membrane suspension. Primary antibodies were directed against GC-A (left panel) or GC-B (right panel) at a dilution of 1:1000 in both cases. The antibodies detected also the HA-tagged variants. In case of GC-A, the HA-tagged variant was even stronger detected by the polyclonal anti-GC-A antibody. Transfection with the empty vector (pcDNA3.1) or non-transfected HEK293T cells did not react with the antibodies. Ligand dependent increase in GC-activities. **(B)** Regulation of GC-A by ANP and of GC-B **(C)** by CNP.

**Figure 5:**
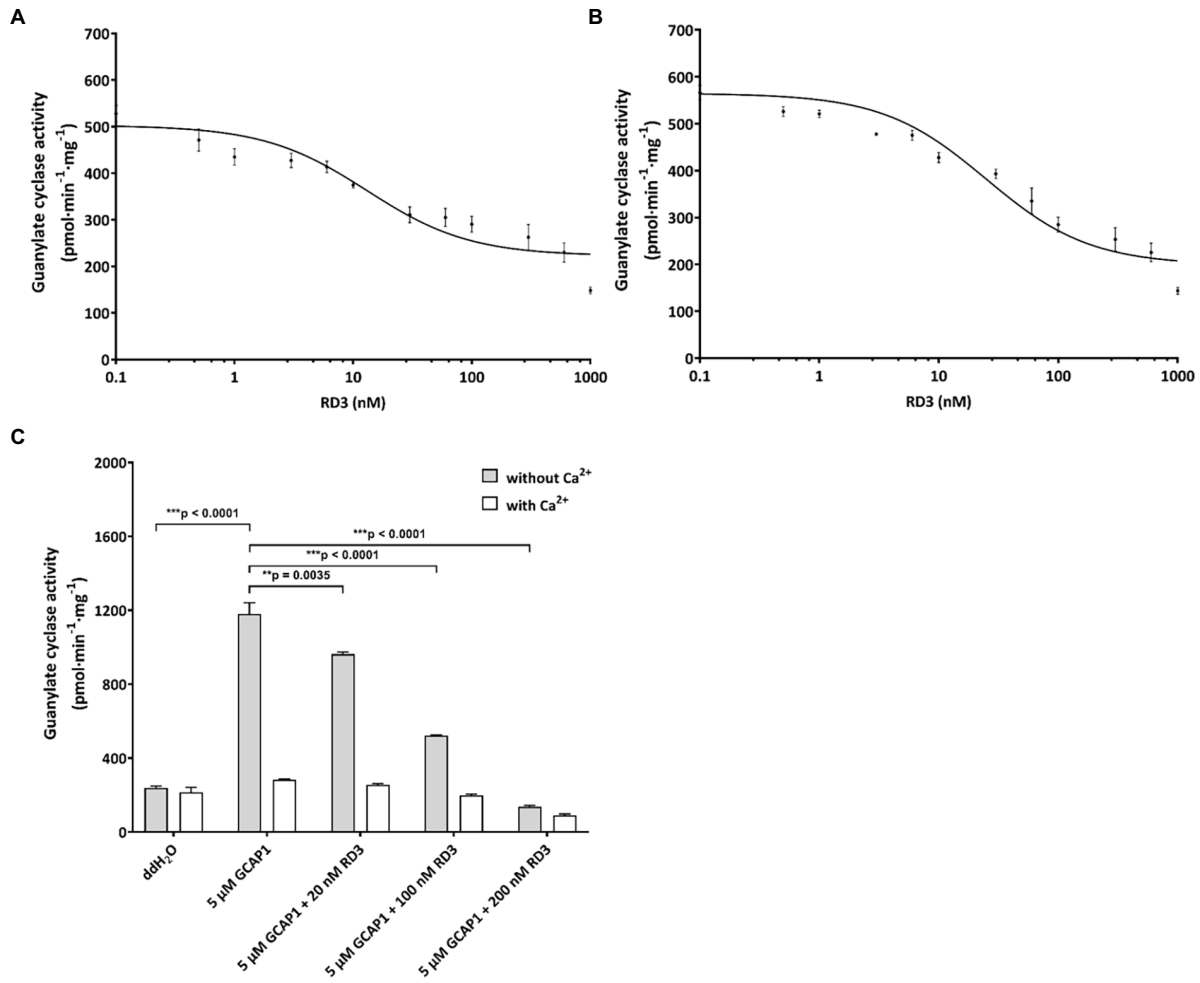
Inhibition of GC variants by RD3. **(A)** GC-A was activated by 2 μM ANP and inhibited by increasing concentrations of RD3. The graph summarizes nine sets of incubations (see Results section). **(B)** GC-B was activated by 2 μM CNP and inhibited by increasing concentrations of RD3 as indicated. The graph summarizes nine sets of incubations. **(C)** Control incubations with photoreceptor GC-E expressed in HEK293T cells that was activated by 5 μM GCAP1 in the absence of Ca^2+^. Adding RD3 at three different concentrations (20 nM, 100 nM, and 200 nM) resulted in decrease in activity being complete between 100 (^***^p = 0.00004511) and 200 nM (^***^p = 0.000007409), *N* = 3. Adding double-distilled water (ddH_2_O) to GC-E in HEK293T cell membranes showed no activation. Analysis of statistics were based on one-way ANOVA followed by Bonferroni’s multiple comparisons test. Data are shown as mean ± SD.

The qRT-PCR data of developmental stages in retina (Figure 1), in different brain areas (Figure 2) and primary cell cultures (Figure 3A) was processed using a one-way analysis of variance (ANOVA) followed by a Bonferroni’s multiple comparisons test. Developmental stages of E14, P0, P20, and P60 were included into gene expression difference analysis of embryonic, birth, adult and elder. Evaluation of cyclase activities regulated by ANP (Figure 4B), CNP, or RD3 (Figures 5A,B) was performed by using the dose response simulation or the inhibition algorithm under nonlinear regression provided by GraphPad Prism 7 (GraphPad Software, San Diego, CA, United States). For the analysis of the functional test of purified RD3 (Figures 3B, 5C) we employed One-Way ANOVA Calculator and Tukey HSD^1^.

## Results

### Expression of natriuretic peptide receptor GCs and RD3 in mouse neuronal tissue

First, we analyzed *rd3*, *Npr1* and *Npr2* transcript levels in the mouse retina at developmental stages P10, P20 and P30 and found high expression levels for *rd3*, but 10-100-fold lower levels for *Npr2* and *Npr1*, respectively (Figure 1). Next, we compared the expression in the retina with those in certain brain regions and observed that *rd3* transcript levels were several orders of magnitude higher in the retina than in brain tissue (compare Figures 1, 2A). For example, the expression of *rd3* in the retina was between 10^−1^ and 10^0^ relative to the β*-actin* expression, but only 10^−4^ to 10^−3^ in the cerebellum, hippocampus, neocortex and the olfactory bulb. These findings confirm the retina being the prominent neuronal tissue of *rd3* localization, but they also showed significant levels of *rd3* transcripts in several brain regions. We tested gene expression pattern of *rd3*, *Npr1* and *Npr2* during development in different mouse brain parts starting from E14 up to P60 (Figure 2). The analysis performed by qRT-PCR included tissue from cerebellum, hippocampus, neocortex, and olfactory bulb. In general, *rd3* showed a lower expression level than *Npr1* and *Npr2* (Figure 2A compared to Figures 2B,C) and reached the highest steady-state expression level in the olfactory bulb between P20 and P60. However, the level was at least one order of magnitude lower than that for *Npr2* in all tested brain tissues (Figure 2C), which reached the highest values between P20 and P60. *Npr1* showed the highest expression at P60 in cerebellum and from P10 onwards in the olfactory bulb. Gene expression of *Npr1* was more dynamic exhibiting a steeper progressive increase in expression levels than *Npr2* and *rd3*. This was particularly visible for patterns observed in cerebellum and olfactory bulb (Figure 2B). Both *Npr* genes types showed lower expression levels in the retina than *rd3*. However, *Npr2* reached normalized expression levels in the retina that were close to those observed in cerebellum and olfactory bulb at P60 (compare Figures 1, 2C).

### RD3 can inhibit hormone activated GC-A and GC-B

Coexpression of *rd3* and natriuretic peptide receptor GC genes in brain tissue led to the next question whether RD3 can regulate the enzymatic activities of membrane bound GC-A and GC-B. The high sequence homology of photoreceptor GCs and natriuretic peptide receptor GCs in the cytoplasmic domains (Goraczniak et al., 1994; Lange et al., 1999) could indicate that membrane bound GCs share similar regulatory features. RD3 is supposed to interact with regions in the cytoplasmic part of photoreceptor GC-E (Peshenko et al., 2011), although the exact binding site has not been determined. To test for any influence of RD3 on GC-A or GC-B activity we tested the impact of purified RD3 on GC activities in a cell culture system. Transient expression of GC-A and GC-B in HEK 293T cells was confirmed by immunoblotting using GC specific antibodies that recognized a single band around 120 kDa (Figure 4A), in agreement with the expectation and the documented molecular masses of membrane bound GCs (Kuhn, 2016). Non-transfected cells (HEK 293T control) or cells transfected with the empty pcDNA3.1 vector showed no GC bands (Figure 4A). HA-tagged variants of GC-A and GC-B were used in positive control transfections. Polyclonal antibodies directed against GC-A or GC-B recognized specifically the HA-tagged GC bands. To our surprise, the tagged variant of GC-A gave a stronger response to the antibody than the non-tagged variant. We have no explanation for this result.

Functional expression of both GCs was tested by monitoring a ligand dependent increase in GC activities. We incubated HEK293T cell membranes containing GC-A or GC-B with increasing concentrations of natriuretic peptides. Both peptides stimulated cGMP production about 10-fold reaching saturation above 4 μM of ANP (activating GC-A) or CNP (activating GC-B; Figures 4B,C). Further controls and test incubations to optimize our assay system included measurements upon GTP dependent cGMP synthesis (Supplementary Figure S1) and a time series resulting in a robust assay system (Materials and Methods) suitable for further studies. Activity tests in the presence and absence of DTT showed no or very negligible differences (Supplementary Figure S1C).

For testing the regulatory impact of RD3 on GC-A or GC-B, we purified RD3 to homogeneity (Supplementary Figure S2) and verified its identity by immunoblotting using two different anti-RD3 antibodies (Supplementary Figure S3). We activated GC-A and GC-B with 2 μM ANP and CNP, respectively, and reconstituted the GC containing HEK 293T cell membranes with increasing concentrations of purified RD3. Inhibition of GC-A was significant, and several replicates (*N* = 9) showed reproducible results yielding a mean IC_50_ value (± SD) of 24 ± 13 nM RD3. A summarizing graph of the experiments is shown in Figure 5A. RD3 had a similar effect upon GC-B activity. The IC_50_ value for half-maximal inhibition was 29 ± 21 nM RD3 (*N* = 9) and a summarizing graph is shown in Figure 5B. However, inhibition was not complete for both GCs (Figures 5A,B) as higher concentrations between 0.5 and 1 μM RD3 did not reach a complete inhibition. Instead, the activity of GC-A and GC-B remained at a constant level (Figures 5A,B). We can exclude by immunoblotting any presence of photoreceptor GC-E in HEK293T cell membranes expressing either GC-A or GC-B (Supplementary Figure S4). This inhibitory feature differed significantly from the effect of RD3 upon photoreceptor specific GC-E (Figure 5C; Peshenko et al., 2011; Wimberg et al., 2018b). Since RD3 is a notoriously unstable protein, we controlled the functional activity of purified RD3 in a well-established assay system. HEK293T cell membranes that stably expressed photoreceptor GC-E were activated in the presence of 5 μM GCAP1. Addition of RD3 inhibited GCAP1 stimulated GC-E activity to a similar extent as reported in the literature (Peshenko et al., 2011; Wimberg et al., 2018b; Figure 5C). This confirmed that RD3 was active and controlled the activity of GC-A and GC-B in a similar, but not identical manner to GC-E.

### Regulation of GC-A and GC-B by RD3 in astrocytes

Extending our analysis to cultured neurons, astrocytes, and microglia, revealed highest expression of *Npr2* in neurons and astrocytes, but also quite high expression of *rd3* in astrocytes (Figure 3A). The relative high transcript levels of *Npr2* and *rd3* in astrocytes led us investigate and compare regulatory features of GC-A and GC-B in primary cultures of astrocytes. Endogenous levels of GC activities around 400 pmol × min^−1^ × mg^−1^ resulted mainly from both GC-A and GC-B as seen in Figure 3 under conditions where no exogenous natriuretic peptides were added (ddH_2_O). Adding natriuretic peptides (ANP and CNP) increased the activities at least twofold. The presence of 30 or 60 nM exogenously added RD3 inhibited the ANP or CNP stimulated GC activity leaving only a basal GC activity level as in the non-stimulated case (Figure 3B). These results showed that endogenously expressed GC-A and GC-B in primary astrocyte cell culture are sensitive to RD3 confirming our results with recombinant GC constructs and pointing to a potential physiologically relevant regulation of natriuretic peptide activated GCs by RD3.

## Discussion

The presence of RD3 in non-retinal tissue seems enigmatic because we lack essential information about its cellular target(s) for defining possible physiological roles. On the other hand, the primary expression site of *rd3* in the mammalian retina (Azadi et al., 2010; Wimberg et al., 2018a; Dizhoor et al., 2019; Plana-Bonamaisó et al., 2020; Figure 1 in this study) well supports several functional roles of RD3 in the retina. For example, RD3 inhibits the activated form of photoreceptor GC-E and GC-F, it is involved in trafficking processes from photoreceptor inner to outer segments and might regulate the nucleotide cycle in photoreceptor cells. In the present work, we investigated the expression pattern of *rd3* in brain tissue and analyzed the enzymatic features of possible target enzymes. The central finding of our study is that RD3 can inhibit two hormone-stimulated GCs, namely GC-A and GC-B indicating a new regulatory feature of these hormone receptors.

Earlier work identified natriuretic peptides and their receptors GC-A and GC-B (or natriuretic peptide receptors) in mammalian retinae using expression and cloning studies (Fernandez-Durango et al., 1989; Kutty et al., 1992; Duda et al., 1993). Subsequent studies performed in various vertebrate species showed the expression of natriuretic peptides and/or their receptors in retinal bipolar, retinal Müller cells, amacrine and ganglion cells (Spreca et al., 1999; Blute et al., 2000; Yu et al., 2006; Cao and Yang, 2007; Abdelalim et al., 2008; Xu et al., 2010). Thus, there is evidence for *Npr1* and *Npr2* expression in retina tissue, but it was unclear so far how the expression level relates to protein levels with a critical function in retinal physiology. In the present study, we compared the transcript levels of *rd3* with those of *Npr1* and *Npr2* and found a relatively high expression level of *Npr2* in the retina. The level is about one order of magnitude higher than that of *Npr1*, but about one order of magnitude lower than that of *rd3*.

Natriuretic peptides and their receptors are involved in various physiological processes in the retina. For example, natriuretic peptides are involved in dopaminergic and cholinergic signaling in amacrine cells (Abdelalim et al., 2008), and Yu et al. (2006) reported that they modulate GABA-receptor activity in bipolar cells and strong immunolabeling of GC-A and GC-B in the outer plexiform layer (OPL). RD3 mainly localizes in inner segments of photoreceptor cells, but in addition expresses in the OPL (Azadi et al., 2010; Wimberg et al., 2018a; Dizhoor et al., 2019). Natriuretic peptide signaling *via* GC-A or GC-B leads to an increase in cGMP targeting, for example cyclic nucleotide-gated ion channels or cGMP dependent protein kinase (PKG). The latter had been discussed as part of a signaling pathway mediating the suppression of GABA-receptor current by BNP (Yu et al., 2006). Thus, RD3 could be involved in balancing the cGMP concentration by inhibiting GC-A or GC-B in the OPL, but high resolution immunohistochemistry of GC-A, GC-B and RD3 to support such a physiological role is missing so far.

Recent studies reported expression of RD3 on the transcript and protein level in several organs and tissues including brain (Khan et al., 2015; Aravindan et al., 2017), but the expression levels appear significantly lower than in the retina. We found that expression of *rd3* is more than 100-fold higher in the retina than in different brain parts (compare Figures 1, 2), which was in broad agreement with the report by Aravindan et al. (2017), who reported significant, but modest or low expression in human cerebellum and olfactory bulb in comparison to retinal expression. When we determined the relative expression levels of *rd3*, *Npr1* and *Npr2* during mice development, it became apparent that the expression of *Npr2* is stronger in all analyzed brain parts than those of *rd3* and *Npr1*. Our findings are consistent with reports showing *Npr2* mRNA expressing cell populations in neocortex, hippocampus, and olfactory bulb (Herman et al., 1996). GC-B has a critical role in the bifurcation of axons during development (Schmidt et al., 2018) extending previous observations that the natriuretic peptide systems play roles in regulating neural development (DiCicco-Bloom et al., 2004; Müller et al., 2009). Collectively, these findings support the notion that CNP is a prominent regulatory factor in the nervous system (Kuhn, 2016; Regan et al., 2021).

Therefore, inhibition of GC-A or GC-B by RD3 in brain tissue might be a critical regulatory feature. We observed stimulation of GC activities by ANP and CNP in astrocytes (Figure 3B) that was broadly consistent with a previous investigation that correlates ligand binding to cGMP accumulation by ANP and CNP (Deschepper and Picard, 1994). We here show that RD3 inhibited both GCs in astrocytes demonstrating that RD3 can exert its effects in a primary cell culture. Although this combination of *in situ* and *in vitro* studies provides only circumstantial evidence for RD3 regulating GC-A and GC-B activities, it might already indicate an impact on the development of retinopathies. In astrocytes, the ANP/GC-A/cGMP signaling counteracts neovascularization in proliferative retinopathies (Burtenshaw and Cahill, 2020; Špiranec Spes et al., 2020). Any inhibitory effect of RD3 would therefore reinforce the development of angiogenic dependent retinopathies indicating how critical the expression level of RD3 is.

Trigger events that up-or downregulate RD3 levels apparently facilitate protein or cell dysfunction. For example, previous work showed that RD3 is downregulated or lost in neuroblastoma cells that remained resistant to multi-modal clinical therapy (Somasundaram et al., 2019). Furthermore, a previous study on the gene expression pattern in the *rd3* mouse, an animal model of congenital blindness with low or no *rd3* expression, reported that more than 1,000 genes are differentially regulated (Cheng and Molday, 2013). An annotation of these genes indicated the involvement of different biochemical pathways including phototransduction, metabolism and a variety of signaling processes. These studies collectively showed that a loss of RD3 is a critical factor in different pathophysiological contexts. It could have an effect on the expression pattern of other proteins in signaling pathways and thereby facilitate tumor development.

In summary, RD3 seems to control the activities of GC-A and GC-B in retinal and non-retinal tissue. These features could be critical for transport processes from the ER to the plasma membrane, but might be involved in different cellular scenarios, where a tight control of intracellular cGMP levels is essential for cell function and survival.

## Data availability statement

The original contributions presented in the study are included in the article/Supplementary material, further inquiries can be directed to the corresponding author.

## Ethics statement

All protocols were in accordance with the German Animal Protection Law and were approved by the local ethics body of Mecklenburg-Western Pomerania (LALLF) and Lower Saxony (LAVES).

## Author contributions

YC, AB, and K-WK designed the study and analyzed the data. YC performed the experiments. K-WK wrote the first draft of the manuscript. YC and AB contributed to writing of the manuscript. All authors contributed to the article and approved the submitted version.

## Funding

This work was supported by a grant from the Deutsche Forschungsgemeinschaft to K-WK (GRK 1885/2) and an intramural research funding of the Faculty VI, School of Medicine and Health Sciences at the University of Oldenburg to AB and K-WK.

## Conflict of interest

The authors declare that the research was conducted in the absence of any commercial or financial relationships that could be construed as a potential conflict of interest.

## Publisher’s note

All claims expressed in this article are solely those of the authors and do not necessarily represent those of their affiliated organizations, or those of the publisher, the editors and the reviewers. Any product that may be evaluated in this article, or claim that may be made by its manufacturer, is not guaranteed or endorsed by the publisher.

## Footnotes

^1^ https://www.statskingdom.com/180Anova1way.html

## References

[ref1] Abdelalim E. M. Masuda C. Tooyama I. (2008). Expression of natriuretic peptide-activated guanylate cyclases by cholinergic and dopaminergic amacrine cells of the rat retina. Peptides 29, 622–628. doi: 10.1016/j.peptides.2007.11.021, PMID: 18192083

[ref2] Aravindan S. Somasundaram D. B. Kam K. L. Subramanian K. Yu Z. Herman T. S.. (2017). Retinal degeneration protein 3 (RD3) in normal human tissues: novel insights. Sci. Rep. 7:13154. doi: 10.1038/s41598-017-13337-9, PMID: 29030614 PMC5640666

[ref3] Azadi S. Molday L. L. Molday R. S. (2010). RD3, the protein associated with Leber congenital amaurosis type 12, is required for guanylate cyclase trafficking in photoreceptor cells. Proc. Natl. Acad. Sci. U. S. A. 107, 21158–21163. doi: 10.1073/pnas.1010460107, PMID: 21078983 PMC3000275

[ref4] Barmashenko G. Buttgereit J. Herring N. Bader M. Ozcelik C. Manahan-Vaughan D.. (2014). Regulation of hippocampal synaptic plasticity thresholds and changes in exploratory and learning behavior in dominant negative NPR-B mutant rats. Front. Mol. Neurosci. 7:95. doi: 10.3389/fnmol.2014.00095, PMID: 25520616 PMC4249455

[ref5] Blute T. A. Lee H. K. Huffmaster T. Haverkamp S. Eldred W. D. (2000). Localization of natriuretic peptides and their activation of particulate guanylate cyclase and nitric oxide synthase in the retina. J. Comp. Neurol. 424, 689–700. PMID: 10931490

[ref6] Burtenshaw D. Cahill P. A. (2020). Natriuretic peptides and the regulation of retinal neovascularization. Arterioscler. Thromb. Vasc. Biol. 40, 7–10. doi: 10.1161/ATVBAHA.119.313566, PMID: 31869266

[ref7] Cao L. H. Yang X. L. (2007). Natriuretic peptide receptor-a is functionally expressed on bullfrog retinal Müller cells. Brain Res. Bull. 71, 410–415. doi: 10.1016/j.brainresbull.2006.10.010, PMID: 17208659

[ref8] Cheng C. L. Molday R. S. (2013). Changes in gene expression associated with retinal degeneration in the rd3 mouse. Mol. Vis. 19, 955–969. PMID: 23687432 PMC3654844

[ref60] Cideciyan A. V. (2010). Leber congenital amaurosis due to RPE65 mutations and its treatment with gene therapy. Prog. Retin. Eye Res. 29, 398–427. doi: 10.1016/j.preteyeres 20399883 PMC2903652

[ref9] Decker J. M. Wójtowicz A. M. Bartsch J. C. Liotta A. Braunewell K. H. Heinemann U.. (2010). C-type natriuretic peptide modulates bidirectional plasticity in hippocampal area CA1 in vitro. Neuroscience 169, 8–22. doi: 10.1016/j.neuroscience.2010.04.064, PMID: 20438814

[ref10] Deschepper C. F. Picard S. (1994). Effects of C-type natriuretic peptide on rat astrocytes: regional differences and characterization of receptors. J. Neurochem. 62, 1974–1982. doi: 10.1046/j.1471-4159.1994.62051974.x, PMID: 7908948

[ref11] DiCicco-Bloom E. Lelièvre V. Zhou X. Rodriguez W. Tam J. Waschek J. A. (2004). Embryonic expression and multifunctional actions of the natriuretic peptides and receptors in the developing nervous system. Dev. Biol. 271, 161–175. doi: 10.1016/j.ydbio.2004.03.028, PMID: 15196958

[ref12] Dizhoor A. M. Olshevskaya E. V. Peshenko I. V. (2019). Retinal guanylyl cyclase activation by calcium sensor proteins mediates photoreceptor degeneration in an rd3 mouse model of congenital human blindness. J. Biol. Chem. 294, 13729–13739. doi: 10.1074/jbc.RA119.009948, PMID: 31346032 PMC6746453

[ref13] Dizhoor A. M. Olshevskaya E. V. Peshenko I. V. (2021). Retinal degeneration-3 protein promotes photoreceptor survival by suppressing activation of guanylyl cyclase rather than accelerating GMP recycling. J. Biol. Chem. 296:100362. doi: 10.1016/j.jbc.2021.100362, PMID: 33539922 PMC8047982

[ref14] Dizhoor A. M. Peshenko I. V. (2021). Regulation of retinal membrane guanylyl cyclase (RetGC) by negative calcium feedback and RD3 protein. Pflugers Arch. 473, 1393–1410. doi: 10.1007/s00424-021-02523-4, PMID: 33537894 PMC8329130

[ref15] Duda T. Goraczniak R. M. Sitaramayya A. Sharma R. K. (1993). Cloning and expression of an ATP-regulated human retina C-type natriuretic factor receptor guanylate cyclase. Biochemistry 32, 1391–1395. doi: 10.1021/bi00057a001, PMID: 7679284

[ref16] Fernandez-Durango R. Sanchez D. Gutkowska J. Carrier F. Fernandez-Cruz A. (1989). Identification and characterization of atrial natriuretic factor receptors in the rat retina. Life Sci. 44, 1837–1846. doi: 10.1016/0024-3205(89)90301-9, PMID: 2544774

[ref17] Friedman J. S. Chang B. Kannabiran C. Chakarova C. Singh H. P. Jalali S.. (2006). Premature truncation of a novel protein, RD3, exhibiting subnuclear localization is associated with retinal degeneration. Am. J. Hum. Genet. 79, 1059–1070. doi: 10.1086/510021, PMID: 17186464 PMC1698706

[ref18] Goraczniak R. M. Duda T. Sitaramayya A. Sharma R. K. (1994). Structural and functional characterization of the rod outer segment membrane guanylate cyclase. Biochem. J. 302, 455–461. doi: 10.1042/bj3020455, PMID: 7916565 PMC1137250

[ref19] Gross I. Tschigor T. Salman A. L. Yang F. Luo J. Vonk D.. (2022). Systematic expression analysis of plasticity-related genes in mouse brain development brings PRG4 into play. Dev. Dyn. 251, 714–728. doi: 10.1002/dvdy.428, PMID: 34622503

[ref20] Herman J. P. Dolgas C. M. Rucker D. Langub M. C. (1996). Localization of natriuretic peptide-activated guanylate cyclase mRNAs in the rat brain. J. Comp. Neurol. 369, 165–187. doi: 10.1002/(SICI)1096-9861(19960527)369, PMID: 8726993

[ref21] Jacobson S. G. Cideciyan A. V. Ho A. C. Roman A. J. Wu V. Garafalo A. V.. (2022). Night vision restored in days after decades of congenital blindness. iScience 25:105274. doi: 10.1016/j.isci.2022.105274, PMID: 36274938 PMC9579015

[ref22] Khan F. H. Pandian V. Ramraj S. K. Aravindan S. Natarajan M. Azadi S.. (2015). RD3 loss dictates high-risk aggressive neuroblastoma and poor clinical outcomes. Oncotarget 6, 36522–36534. doi: 10.18632/oncotarget.5204, PMID: 26375249 PMC4742193

[ref23] Koch K.-W. Dell'Orco D. (2015). Protein and signaling networks in vertebrate photoreceptor cells. Front. Mol. Neurosci. 8:67. doi: 10.3389/fnmol.2015.00067, PMID: 26635520 PMC4646965

[ref24] Kuhn M. (2016). Molecular physiology of membrane guanylyl cyclase receptors. Physiol. Rev. 96, 751–804. doi: 10.1152/physrev.00022.2015 27030537

[ref25] Kutty R. K. Fletcher R. T. Chader G. J. Krishna G. (1992). Expression of guanylate cyclase-a mRNA in the rat retina: detection using polymerase chain reaction. Biochem. Biophys. Res. Commun. 182, 851–857. doi: 10.1016/0006-291x(92)91810-d, PMID: 1370893

[ref65] Lange C. Duda T. Beyermann M. Sharma R. K. Koch K. W. (1999). Regions in vertebrate photoreceptor guanylyl cyclase ROS-GC1 involved in Ca(2+)-dependent regulation by guanylyl cyclase-activating protein GCAP-1. FEBS Lett. 460, 27–31. doi: 10.1016/s0014-5793(99)01312-5., PMID: 10571055

[ref26] Molday L. L. Djajadi H. Yan P. Szczygiel L. Boye S. L. Chiodo V. A.. (2013). RD3 gene delivery restores guanylate cyclase localization and rescues photoreceptors in the Rd3 mouse model of Leber congenital amaurosis 12. Hum. Mol. Genet. 22, 3894–3905. doi: 10.1093/hmg/ddt244, PMID: 23740938 PMC3766183

[ref27] Müller D. Hida B. Guidone G. Speth R. C. Michurina T. V. Enikolopov G.. (2009). Expression of guanylyl cyclase (GC)-a and GC-B during brain development: evidence for a role of GC-B in perinatal neurogenesis. Endocrinology 150, 5520–5529. doi: 10.1210/en.2009-0490, PMID: 19837875

[ref28] Olcese J. Majora C. Stephan A. Müller D. (2002). Nocturnal accumulation of cyclic 3′,5′-guanosine monophosphate (cGMP) in the chick pineal organ is dependent on activation of guanylyl cyclase-B. J. Neuroendocrinol. 14, 14–18. doi: 10.1046/j.0007-1331.2001.00732.x, PMID: 11903808

[ref29] Pandey K. N. (2021). Molecular signaling mechanisms and function of natriuretic peptide receptor-a in the pathophysiology of cardiovascular homeostasis. Front. Physiol. 12:693099. doi: 10.3389/fphys.2021.693099, PMID: 34489721 PMC8416980

[ref30] Perrault I. Estrada-Cuzcano A. Lopez I. Kohl S. Li S. Testa F.. (2013). Union makes strength: a worldwide collaborative genetic and clinical study to provide a comprehensive survey of RD3 mutations and delineate the associated phenotype. PLoS One 8:e51622. doi: 10.1371/journal.pone.0051622, PMID: 23308101 PMC3538699

[ref31] Peshenko I. V. Dizhoor A. M. (2020). Two clusters of surface-exposed amino acid residues enable high-affinity binding of retinal degeneration-3 (RD3) protein to retinal guanylyl cyclase. J. Biol. Chem. 295, 10781–10793. doi: 10.1074/jbc.RA120.013789, PMID: 32493772 PMC7397094

[ref32] Peshenko I. V. Olshevskaya E. V. Dizhoor A. M. (2016). Functional study and mapping sites for interaction with the target enzyme in retinal degeneration 3 (RD3) protein. J. Biol. Chem. 291, 19713–19723. doi: 10.1074/jbc.M116.742288, PMID: 27471269 PMC5016703

[ref33] Peshenko I. V. Olshevskaya E. V. Dizhoor A. M. (2021). Retinal degeneration-3 protein attenuates photoreceptor degeneration in transgenic mice expressing dominant mutation of human retinal guanylyl cyclase. J. Biol. Chem. 297:101201. doi: 10.1016/j.jbc.2021.101201, PMID: 34537244 PMC8517212

[ref34] Peshenko I. V. Olshevskaya E. V. Savchenko A. B. Karan S. Palczewski K. Baehr W.. (2011). Enzymatic properties and regulation of the native isozymes of retinal membrane guanylyl cyclase (RetGC) from mouse photoreceptors. Biochemistry 50, 5590–5600. doi: 10.1021/bi200491b, PMID: 21598940 PMC3127287

[ref35] Plana-Bonamaisó A. López-Begines S. Andilla J. Fidalgo M. J. Loza-Alvarez P. Estanyol J. M.. (2020). GCAP neuronal calcium sensor proteins mediate photoreceptor cell death in the rd3 mouse model of LCA12 congenital blindness by involving endoplasmic reticulum stress. Cell Death Dis. 11:62. doi: 10.1038/s41419-020-2255-0, PMID: 31980596 PMC6981271

[ref36] Preising M. N. Hausotter-Will N. Solbach M. C. Friedburg C. Rüschendorf F. Lorenz B. (2012). Mutations in RD3 are associated with an extremely rare and severe form of early onset retinal dystrophy. Invest. Ophthalmol. Vis. Sci. 53, 3463–3472. doi: 10.1167/iovs.12-9519, PMID: 22531706 PMC3390007

[ref37] Regan J. T. Mirczuk S. M. Scudder C. J. Stacey E. Khan S. Worwood M.. (2021). Sensitivity of the natriuretic peptide/cGMP system to hyperammonaemia in rat C6 glioma cells and GPNT brain endothelial cells. Cells 10:398. doi: 10.3390/cells10020398, PMID: 33672024 PMC7919485

[ref38] Rollín R. Mediero A. Roldán-Pallarés M. Fernández-Cruz A. Fernández-Durango R. (2004). Natriuretic peptide system in the human retina. Mol. Vis. 10, 15–22. PMID: 14737067

[ref39] Schmidt H. Dickey D. M. Dumoulin A. Octave M. Robinson J. W. Kühn R.. (2018). Regulation of the natriuretic peptide receptor 2 (Npr2) by phosphorylation of juxtamembrane serine and threonine residues is essential for bifurcation of sensory axons. J. Neurosci. 38, 9768–9780. doi: 10.1523/JNEUROSCI.0495-18.2018, PMID: 30249793 PMC6222061

[ref40] Sharma R. K. (2010). Membrane guanylate cyclase is a beautiful signal transduction machine: overview. Mol. Cell. Biochem. 334, 3–36. doi: 10.1007/s11010-009-0336-6, PMID: 19957201

[ref41] Sharma R. K. Duda T. Makino C. L. (2016). Integrative signaling networks of membrane guanylate cyclases: biochemistry and physiology. Front. Mol. Neurosci. 9:83. doi: 10.3389/fnmol.2016.00083, PMID: 27695398 PMC5023690

[ref42] Sharon D. Wimberg H. Kinarty Y. Koch K.-W. (2018). Genotype-functional-phenotype correlations in photoreceptor guanylate cyclase (GC-E) encoded by GUCY2D. Prog. Retin. Eye Res. 63, 69–91. doi: 10.1016/j.preteyeres.2017.10.003, PMID: 29061346

[ref43] Somasundaram D. B. Subramanian K. Aravindan S. Yu Z. Natarajan M. Herman T.. (2019). De novo regulation of RD3 synthesis in residual neuroblastoma cells after intensive multi-modal clinical therapy harmonizes disease evolution. Sci. Rep. 9:11766. doi: 10.1038/s41598-019-48034-2, PMID: 31409909 PMC6692366

[ref44] Špiranec Spes K. Hupp S. Werner F. Koch F. Völker K. Krebes L.. (2020). Natriuretic peptides attenuate retinal pathological neovascularization via cyclic guanosine monophosphate signaling in pericytes and astrocytes. Arterioscler. Thromb. Vasc. Biol. 40, 159–174. doi: 10.1161/ATVBAHA.119.313400, PMID: 31619060

[ref45] Spreca A. Giambanco I. Rambotti M. G. (1999). Ultracytochemical study of guanylate cyclases a and B in light-and dark-adapted retinas. Histochem. J. 31, 477–483. doi: 10.1023/a:1003712110751, PMID: 10475575

[ref46] Sulmann S. Kussrow A. Bornhop D. J. Koch K.-W. (2017). Label-free quantification of calcium-sensor targeting to photoreceptor guanylate cyclase and rhodopsin kinase by backscattering interferometry. Sci. Rep. 7:45515. doi: 10.1038/srep45515, PMID: 28361875 PMC5374524

[ref47] Wimberg H. Janssen-Bienhold U. Koch K.-W. (2018a). Control of the nucleotide cycle in photoreceptor cell extracts by retinal degeneration protein 3. Front. Mol. Neurosci. 11:52. doi: 10.3389/fnmol.2018.00052, PMID: 29515371 PMC5826319

[ref48] Wimberg H. Lev D. Yosovich K. Namburi P. Banin E. Sharon D.. (2018b). Photoreceptor guanylate cyclase (GUCY2D) mutations cause retinal dystrophies by severe malfunction of Ca2+−dependent cyclic GMP synthesis. Front. Mol. Neurosci. 11:348. doi: 10.3389/fnmol.2018.00348, PMID: 30319355 PMC6167591

[ref49] Xu G. Z. Tian J. Zhong Y. M. Yang X. L. (2010). Natriuretic peptide receptors are expressed in rat retinal ganglion cells. Brain Res. Bull. 82, 188–192. doi: 10.1016/j.brainresbull.2010.03.004, PMID: 20304036

[ref50] Yu Y. C. Cao L. H. Yang X. L. (2006). Modulation by brain natriuretic peptide of GABA receptors on rat retinal ON-type bipolar cells. J. Neurosci. 26, 696–707. doi: 10.1523/JNEUROSCI.3653-05.2006, PMID: 16407567 PMC6674405

[ref51] Zägel P. Dell'Orco D. Koch K.-W. (2013). The dimerization domain in outer segment guanylate cyclase is a Ca2+−sensitive control switch module. Biochemistry 52, 5065–5074. doi: 10.1021/bi400288p, PMID: 23815670

[ref52] Zulliger R. Naash M. I. Rajala R. V. Molday R. S. Azadi S. (2015). Impaired association of retinal degeneration-3 with guanylate cyclase-1 and guanylate cyclase-activating protein-1 leads to leber congenital amaurosis-1. J. Biol. Chem. 290, 3488–3499. doi: 10.1074/jbc.M114.616656, PMID: 25477517 PMC4319016

