# Supplementary material for: Retinal degeneration protein 3 controls membrane guanylate cyclase activities in brain tissue
Source: Front Mol Neurosci. 2022 Dec 21;15:1076430. doi: 10.3389/fnmol.2022.1076430 (PMC9812585; doi:10.3389/fnmol.2022.1076430)
Supplement: Supplementary file 1 [file Data_Sheet_1.docx]

Supplementary Material

**Retinal degeneration protein 3 controls membrane guanylate cyclase activities in brain tissue**

Yaoyu Chen^1,2^, Anja Bräuer^2,3^, Karl-Wilhelm Koch^1,3,*^

^1^Division of Biochemistry, Department of Neuroscience, Carl von Ossietzky University, Oldenburg, Germany

^2^Division of Anatomy, Department of Human Medicine, Carl von Ossietzky University, Oldenburg, Germany

^3^Research Center Neurosensory Science, Carl von Ossietzky University Oldenburg, Oldenburg, Germany

*corresponding author: KWK: Tel.: +49-(0)441-798-3640;

E-mail: [karl.w.koch@uni-oldenburg.de](mailto:karl.w.koch@uni-oldenburg.de)

**Figure S1**

**A**


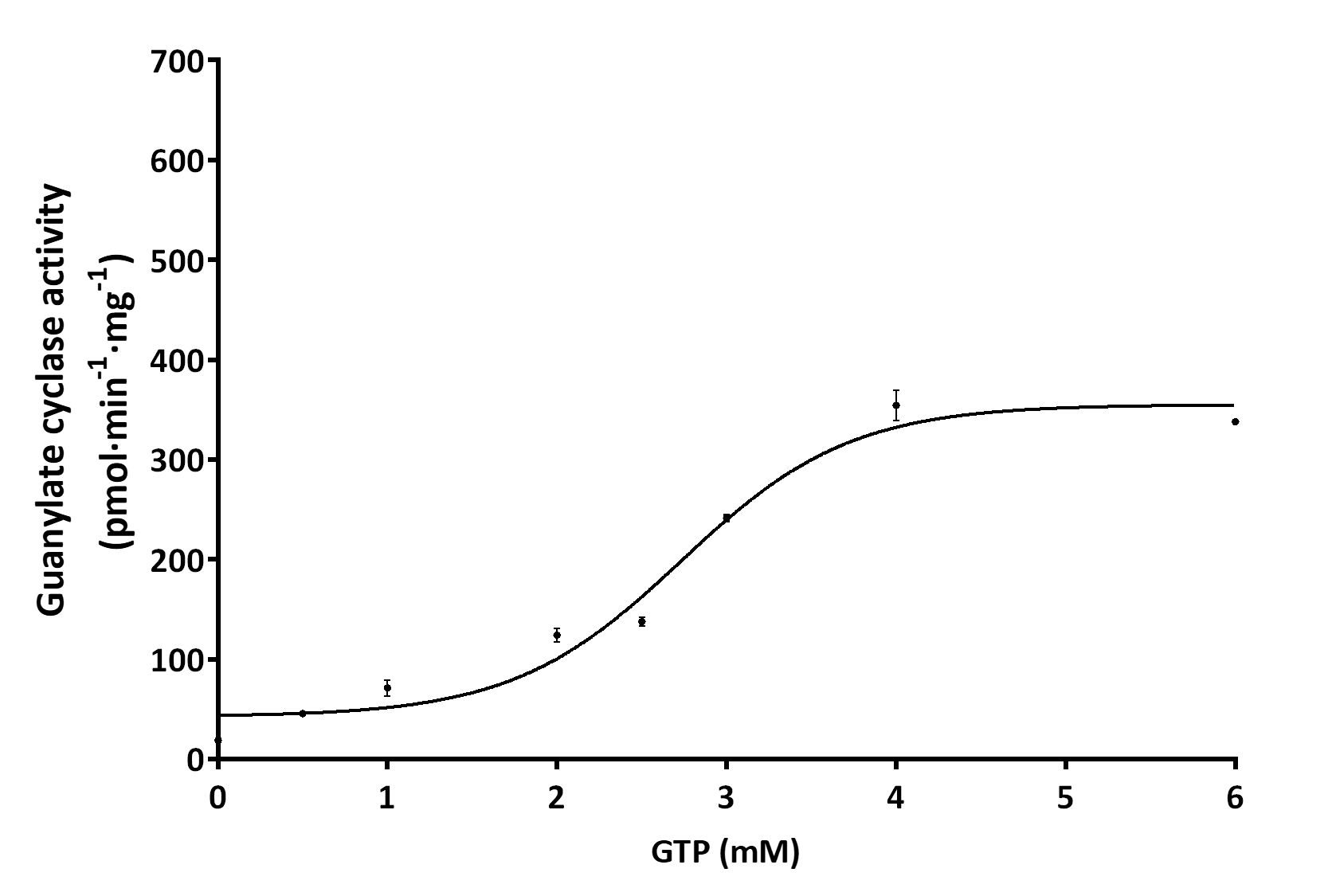


**B**


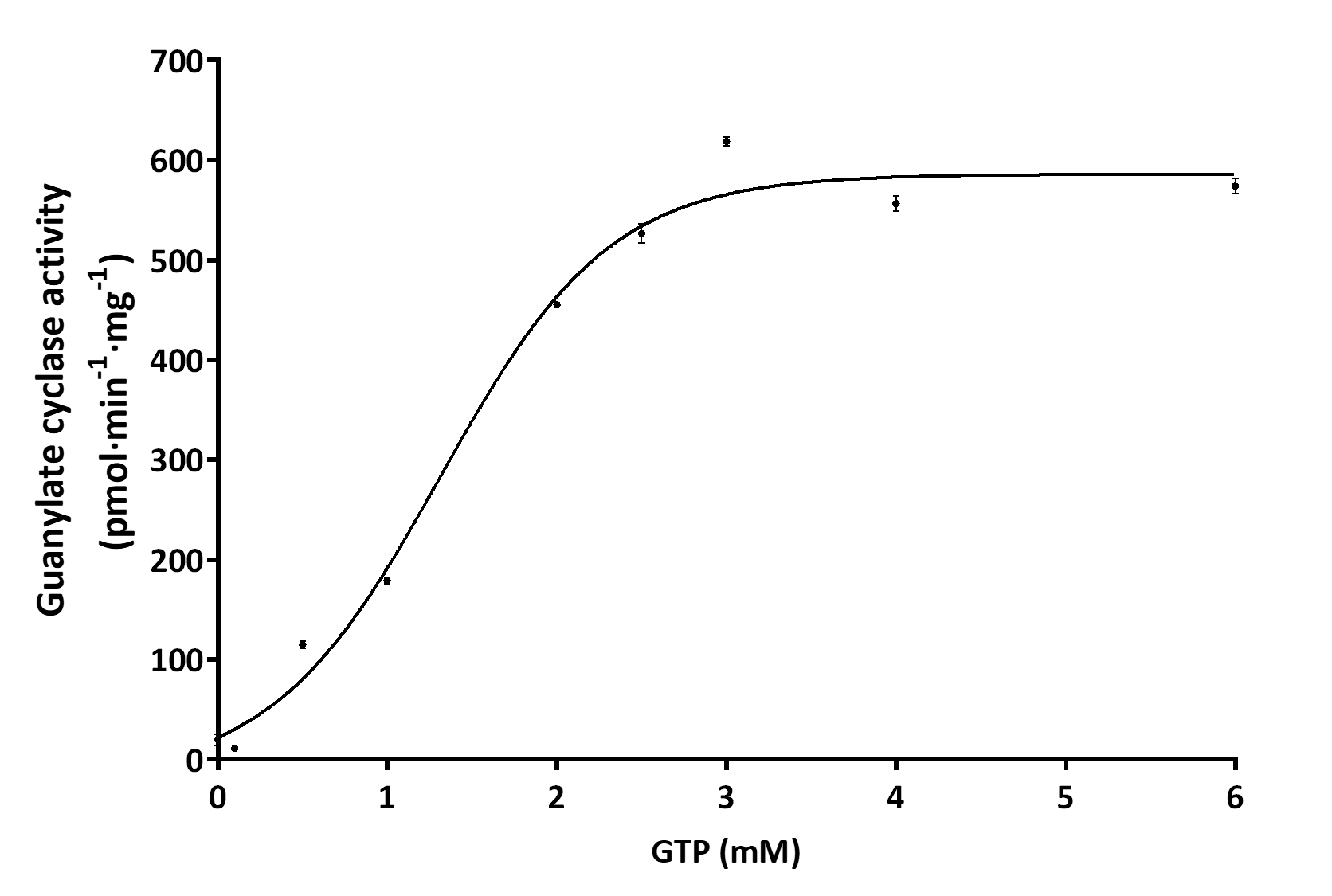


**C**

**
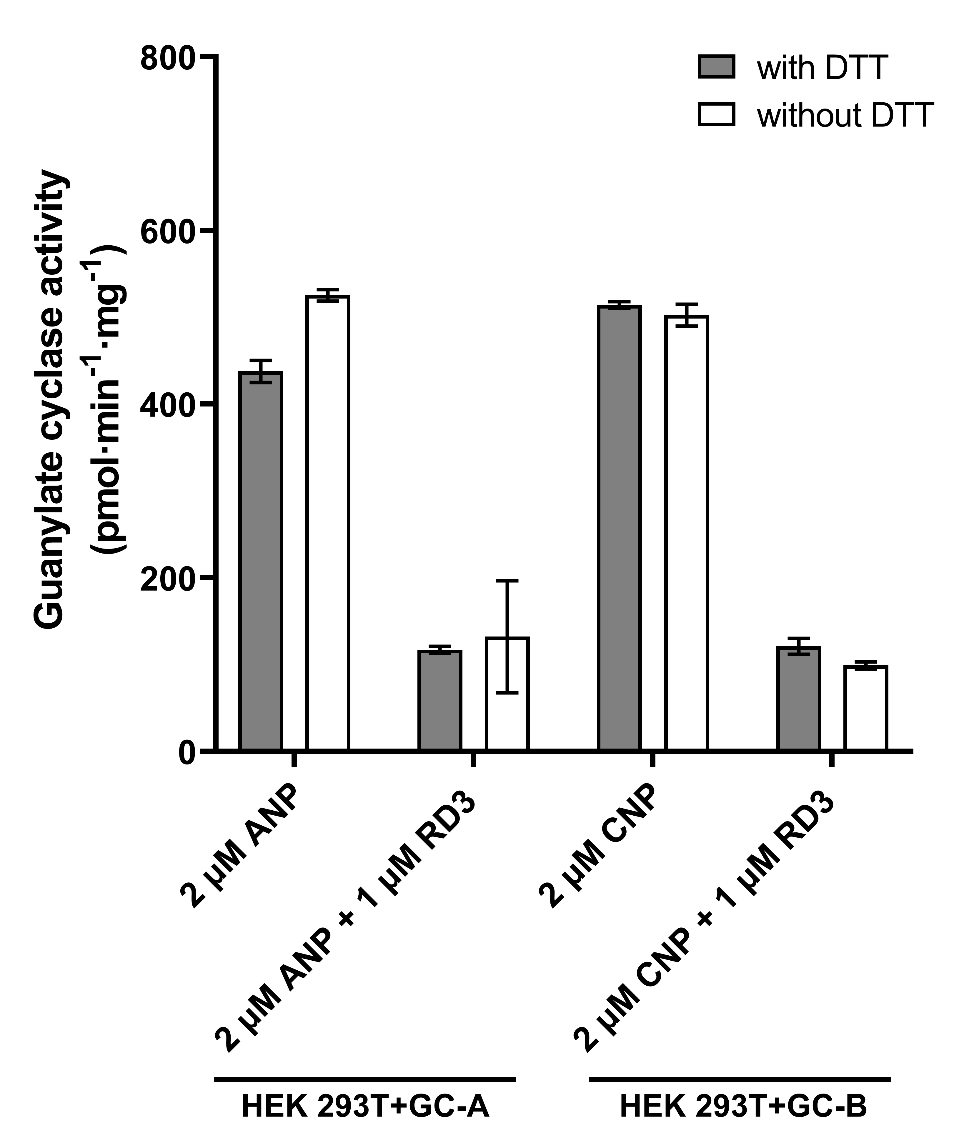
**

**Figure S1.** Enzymatic activity of GC-A (A) and GC-B (B) as a function of the substrate GTP concentration. (C) Comparison of GC activities in the presence and absence of DTT. GC-A was activated by 2 µM ANP, GC-B was activated by 2 µM CNP. Addition of 1 µM RD3 decreased GC-activities (N = 3).

**Figure S2**


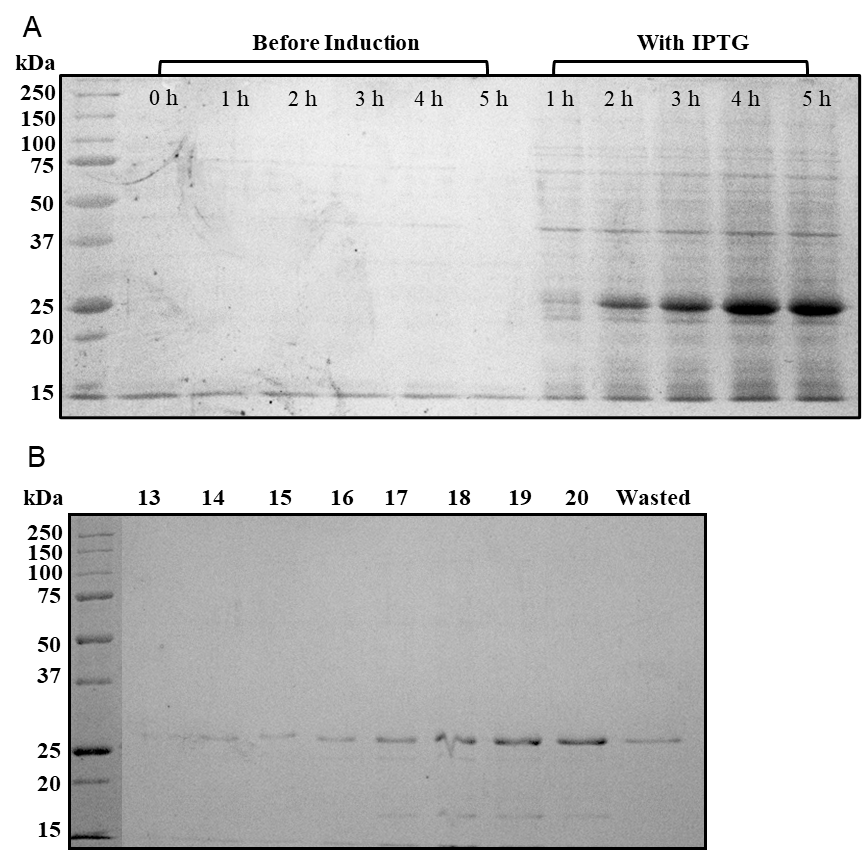


**Figure S2.** Purification of recombinant RD3 analyzed by SDS PAGE and Coomassie Blue staining. A. RD3 protein expression in *E.coli* before and after the IPTG induction. B. Column chromatography yielding purified RD3 by elution from a Ni-NTA column. Collection of purified samples started at fraction 13.

**Figure S3**


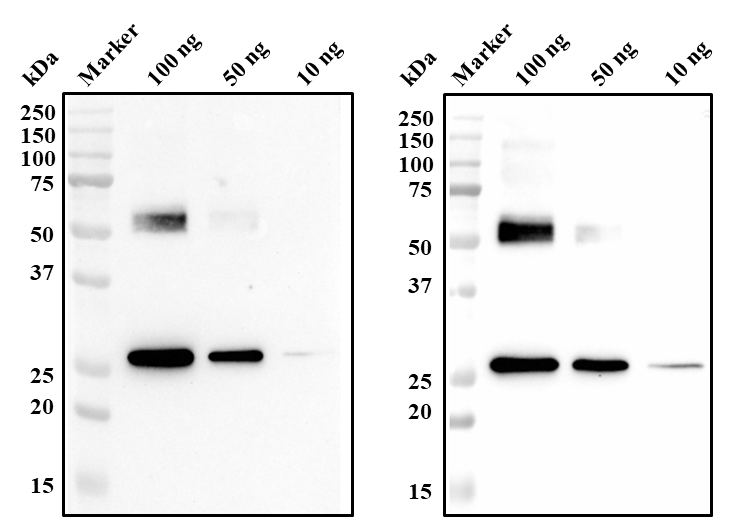


**Figure S3.** Immunodetection of purified RD3. Immunoblots were incubated with **(A)** mouse anti-RD3 antibody (sc-376516, Abcam) or **(B)** rabbit anti-RD3 antibody (PA598582, Thermo) at protein amounts of 10 ng, 50 ng and 100 ng. Monomers (above 25 kDa) and dimers (above 50 kDa) are visible.

**Figure S4**

**
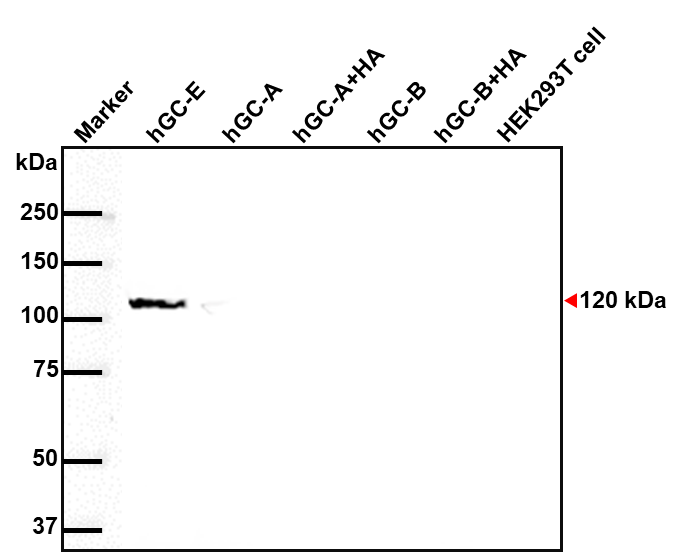
**

**Figure S4.** Immunoblotting for testing expression of GC-E. HEK293T cells transfected with human GC-A or GC-B constructs or not transfected (HEK 293 T) were probed a polyclonal anti-GC-E antibody (GC-E #3, see Zägel et al., 2913) at a dilution of 1:1,500. Secondary antibody was a horse-radish peroxidase-coupled goat anti rabbit at a dilution of 1:10,000. Positive control was a sample of HEK293 T cells expressing human GC-E at 120 kDa as indicated. No signal was observed in cells expressing human GC-A or GC-B.

**Table S1.** Primers for amplification of human GC-A and human GC-B

GC-A: 5’-GCTAGCCCACCATGCCTGGGACCGGC-3’

3’-GAACGAAGATGGTACTCGTCTCTGTTAATT-5’.

GC-B: 5’ -GCTAGCCCACCTGAGTACCTGGCACCGCT-3’,

3’-TGTGACGAAGAGCTTACCTCAT-5’

**Table S2.** Statistics of relative expression levels of *rd3*, *Npr1* and *Npr2* in the retina as indicated. Significant differences are indicated with asterisks (p ≤ 0.05 = *; p ≤ 0.01 = **; p ≤ 0.001 = ***).

| Genes | P10 vs P20 | | P10 vs P30 | | P20 vs P30 | |
| --- | --- | --- | --- | --- | --- | --- |
| *rd3* | * | 0.0422 | ns | 0.0521 | ns | 0.8222 |
| *Npr1* | * | 0.0104 | ns | 0.0538 | ns | 0.0976 |
| *Npr2* | ** | 0.0012 | *** | 0.0006 | ns | 0.2772 |

**Table S3.** Statistics of relative expression levels of RD3, GC-A and GC-B in brain regions as indicated. Significant differences are indicated with asterisks (p ≤ 0.05 = *; p ≤ 0.01 = **; p ≤ 0.001 = ***).

| Genes | Group | Cerebellum | | Hippocampus | | Neocortex | | Olfactory bulbs | |
| --- | --- | --- | --- | --- | --- | --- | --- | --- | --- |
| *rd3* | E14 vs P0 | * | 0.0128 | ns | 0.2425 | ns | 0.7289 | ns | 0.3317 |
|  | P0 vs P20 | ns | 0.5782 | * | 0.0390 | ns | 0.5066 | ns | 0.0508 |
|  | P20 vs P60 | ns | 0.7748 | * | 0.0136 | ns | 0.3098 | ns | 0.4406 |
|  | E14 vs P60 | ns | 0.1129 | * | 0.0173 | ns | 0.1724 | ** | 0.0068 |
| *Npr1* | E14 vs P0 | ns | 0.0578 | ns | 0.5123 | ns | 0.1226 | *** | 0.0009 |
|  | P0 vs P20 | * | 0.0412 | ** | 0.0068 | * | 0.0107 | ns | 0.0670 |
|  | P20 vs P60 | * | 0.0189 | ** | 0.0050 | ** | 0.0624 | ns | 0.3405 |
|  | E14 vs P60 | ** | 0.0014 | ** | 0.0014 | *** | 0.0003 | * | 0.0122 |
| *Npr2* | E14 vs P0 | ns | 0.0578 | ns | 0.1031 | ns | 0.6380 | * | 0.0190 |
|  | P0 vs P20 | ** | 0.0023 | * | 0.0365 | * | 0.0445 | * | 0.0232 |
|  | P20 vs P60 | * | 0.0198 | *** | 0.0009 | ns | 0.1072 | ns | 0.2252 |
|  | E14 vs P60 | ** | 0.0030 | ** | 0.0084 | ns | 0.0552 | ** | 0.0045 |

**Table S4.** Statistics of relative expression levels of *rd3*, *Npr1* and *Npr2* in neurons, astrocytes, and microglia. Significant differences are indicated with asterisks (p ≤ 0.05 = *; p ≤ 0.01 = **; p ≤ 0.001 = ***).

| Group | *rd3* | | *Npr1* | | *Npr2* | |
| --- | --- | --- | --- | --- | --- | --- |
| Neuron vs Astrocyte | * | 0.01426 | ns | 0.40810 | * | 0.01968 |
| Astrocyte vs Microglia | * | 0.01350 | ** | 0.00309 | * | 0.01226 |
| Microglia vs Neuron | ns | 0.39850 | *** | 0.00015 | * | 0.01506 |
