# Supplementary material for: Retinal degeneration protein 3 controls membrane guanylate cyclase activities in brain tissue
Source: Front Mol Neurosci. 2022 Dec 21;15:1076430. doi: 10.3389/fnmol.2022.1076430 (PMC9812585; doi:10.3389/fnmol.2022.1076430)
Supplement: Supplementary file 2 [file Presentation_1.PPTX]

## Slide 1
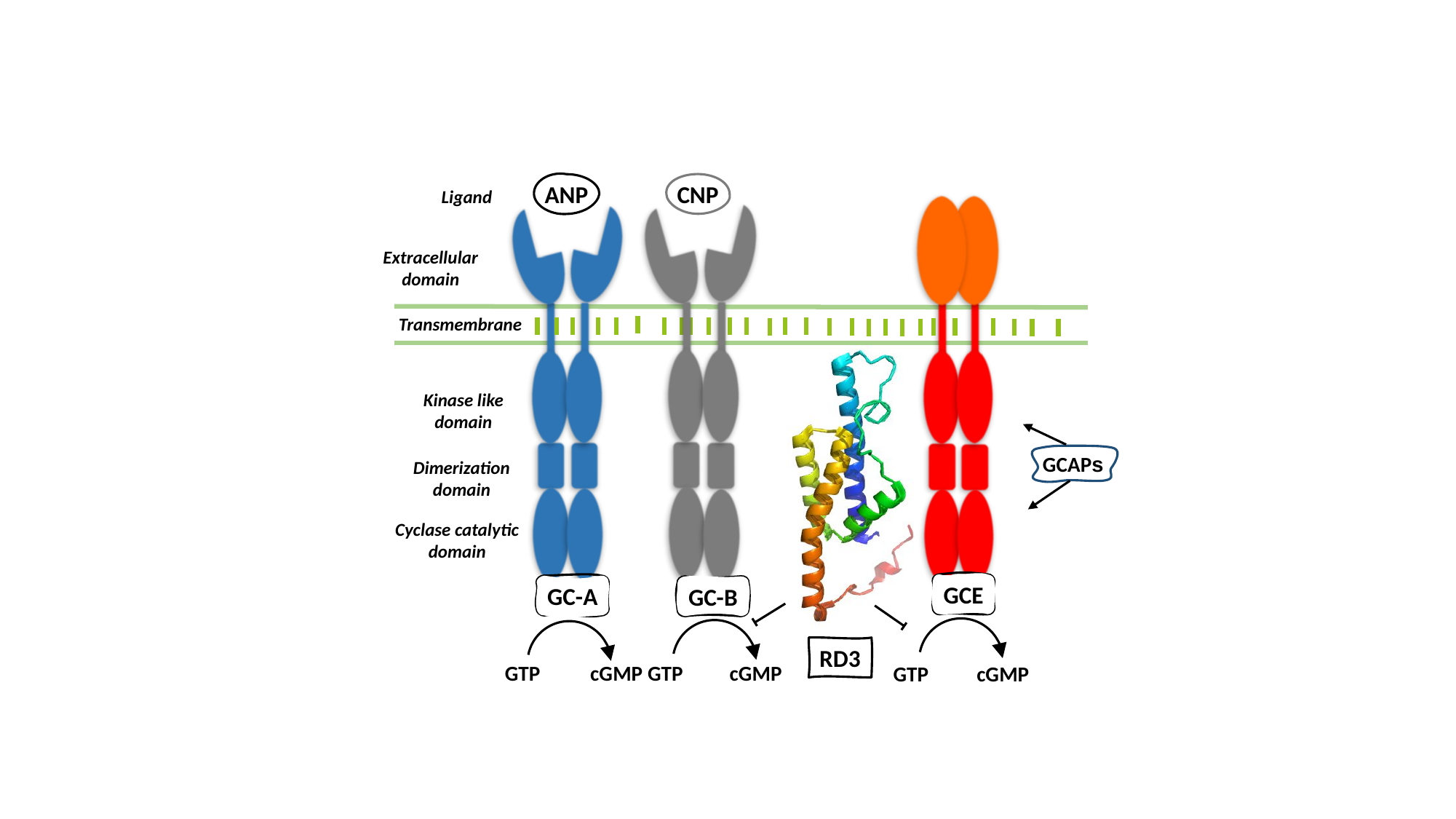

CNP
ANP
Ligand
Extracellular
domain
Transmembrane
Kinase like domain
GCAPs
Dimerization
domain
Cyclase catalytic domain
GCE
GC-A
GC-B
RD3
GTP
cGMP
GTP
cGMP
GTP
cGMP
